# Gingivitis: The Past, the Present, the Future

**DOI:** 10.1111/jre.70041

**Published:** 2025-09-25

**Authors:** Leonardo Trombelli

**Affiliations:** ^1^ Research Centre for the Study of Periodontal and Peri‐Implant Diseases University of Ferrara; Operative Unit of Dentistry Ferrara Italy

**Keywords:** bleeding on probing, diagnosis, gingivitis, personalized dentistry, plaque‐induced gingivitis

Gingivitis is one of the most widespread oral diseases, affecting individuals across all age groups and geographic regions. Its global prevalence is remarkably high: epidemiological surveys consistently show that the majority of adults present with some degree of gingival inflammation at any given time, and nearly everyone will experience it during their lifetime.

Plaque‐induced gingival inflammation lesion is a reversible lesion confined to the supra‐crestal tissues, resulting from the accumulation of a microbial biofilm at the gingival margin and modulated by the host's immune‐inflammatory response.

Although gingivitis lesion does not involve loss of periodontal attachment, its clinical significance is far from negligible. It is the primary cause of bleeding gums, often the earliest sign of compromised gingival health, and it can negatively affect quality of life by causing discomfort, halitosis, and esthetic concerns. Moreover, in susceptible individuals, it may represent a precursor state that, if left unmanaged, can progress to periodontitis, an irreversible and destructive condition.

Over time, our understanding of the gingivitis condition has shifted from a simplistic microbial paradigm to a nuanced model that integrates microbial challenge, host response, and environmental influences, and now toward technology‐enabled, individualized management. Based on (a) the hypothesis that susceptibility to periodontitis and susceptibility to gingivitis may partly share common risk factors and (b) the awareness that the successful treatment of gingivitis represents the primary preventive measure of periodontitis, research efforts should be focused on the effect of genetic, anatomic, and environmental host‐related factors which could be implicated in the pathogenesis of the gingival inflammatory process. In this respect, the identification of factors related to increased susceptibility to gingivitis may help identify, at an early age, subjects at risk of periodontitis.

## 1 The Past

The scientific foundation for the role of dental plaque in the initiation of gingivitis lesions was firmly established in the 1960s through the experimental gingivitis model [1].

In these landmark studies, subjects suspended oral hygiene, allowing plaque to accumulate, which consistently resulted in the onset of clinical inflammation. Upon resumption of effective plaque removal, inflammation resolved completely. This reproducible cause–effect relationship established plaque as the necessary and sufficient etiologic factor in gingivitis, a concept that guided both clinical practice and preventive strategies for decades.

Initially, variations in the severity of gingivitis between individuals were attributed to differences in plaque quantity or composition. However, refined experimental models developed in the 1990s and 2000s at our Research Center at UniFE revealed a more complex reality: even under standardized plaque accumulation, individuals displayed stable and reproducible inflammatory profiles over time [2]. Some subjects consistently exhibited pronounced inflammation (“high responders”) whereas others showed minimal changes (“low responders”) despite similar amounts of plaque deposits [3]. These phenotypic differences persisted across repeated experimental challenges, suggesting a strong biological basis [4, 5].

Subsequent research investigated the determinants of these individual differences. Genetic studies identified associations between certain cytokine gene polymorphisms—such as those in the interleukin‐1 cluster—and heightened inflammatory expression [6]. Analysis of gingival crevicular fluid revealed biomarkers, including interleukin‐1β, calprotectin, and prostaglandin E2, which correlated with clinical signs of inflammation and provided insight into the underlying immunopathology. Other studies explored behavioral and psychosocial influences, noting that acute stress could exacerbate inflammation, but the core responder phenotype appeared largely stable and biologically determined even when a proper oral hygiene regimen was reinstated.

By the early 21st century, this body of evidence had transformed the conceptualization of gingival inflammatory lesions from a uniform consequence of plaque accumulation to a dynamic condition shaped by host susceptibility, immune regulation, and site‐specific environmental factors. This paradigm shift laid the groundwork for precision prevention strategies tailored to an individual's inflammatory profile.

## 2 The Present

Today, plaque‐induced gingivitis is clearly defined within the framework of the 2017 World Workshop on the Classification of Periodontal and Peri‐Implant Diseases and Conditions, where for the first time a set of criteria to identify a “gingivitis case” was established [7]. At the patient level, diagnosis requires bleeding on probing (BoP) at 10% or more of sites, probing depths of 3 mm or less, and no clinical attachment or bone loss. The extent is classified as localized (10%–30% of BoP+ sites) or generalized (> 30% of BoP+ sites). These diagnostic thresholds apply both to intact periodontium and to reduced but stable periodontium without a history of periodontitis, making them broadly applicable in both clinical and epidemiological contexts [8].

BoP remains the cornerstone of diagnosis due to its simplicity, reproducibility, and prognostic value. It also serves as an effective communication tool in patient education, providing tangible evidence of inflammation. Nevertheless, the variability in inflammatory expression observed in experimental models is mirrored in routine practice, where some patients exhibit extensive BoP despite minimal plaque deposits. This reinforces the importance of considering host‐related factors and raises questions about whether certain gingivitis phenotypes carry a higher risk of progression to periodontitis.

The clinical relevance of gingivitis extends beyond the oral cavity. A growing body of research links gingival inflammation to elevated systemic inflammatory markers such as C‐reactive protein and interleukin‐6, suggesting a potential role in systemic disease burden.

Furthermore, patients often report impacts on self‐esteem, social interactions, and daily comfort, highlighting the importance of managing gingivitis not only as a preventive measure against periodontitis but also as a contributor to overall well‐being.

Current management focuses on controlling dental plaque through effective self‐care—brushing, interdental cleaning—and professional interventions when necessary.

Behavioral modification strategies, patient‐centered education, and motivational interviewing have been shown to enhance adherence to oral hygiene regimens.

In this contemporary framework, diagnosis is more than a label; it is the starting point for personalized prevention plans.

## 3 The Future

Looking ahead, the future of gingivitis diagnosis and management will be shaped by advances in digital technologies, precision medicine, and integrated healthcare delivery.

While BoP will remain a reference point, it is inherently subjective and dependent on operator skill, prompting interest in more objective, technology‐assisted tools.

Recent studies have explored the use of digital intraoral scans combined with automated colorimetric analysis to detect gingival inflammation. The analysis of gingival color as captured by intraoral scans has demonstrated a range of performance from acceptable to excellent in distinguishing sites with gingival inflammation (BoP+) [9]. Parallel advances in plaque quantification include two‐dimensional photographic analysis and three‐dimensional full‐arch mapping, providing highly reproducible, examiner‐independent measurements [10]. These visual tools can be integrated into patient consultations, offering compelling feedback that reinforces oral hygiene compliance.

The integration of imaging data with salivary biomarker profiles, microbiome analysis, and genetic risk assessment represents the next frontier in personalized gingivitis care.

Machine learning algorithms could synthesize these inputs to generate individualized risk scores, predict disease trajectories, and recommend tailored preventive interventions.

Moreover, app‐based monitoring systems and wearable biosensors may enable continuous assessment of gingival health, shifting care from episodic dental visits to ongoing, proactive management.

In this envisioned future, gingivitis care will be minimally invasive, data‐driven, and seamlessly integrated into broader preventive healthcare frameworks. Patients will benefit from early detection, personalized education, and continuous support, ultimately reducing the prevalence of gingivitis and its potential progression to periodontitis, thus resulting in an overall benefit for both oral and systemic health.

## Conflicts of Interest

The author declares no conflicts of interest.

## Data Availability

Data sharing not applicable to this article as no datasets were generated or analyzed during the current study.
